# Maize Germplasm Conservation in Southern California’s Urban Gardens: Introduced Diversity Beyond *ex situ* and *in situ* Management

**DOI:** 10.1007/s12231-016-9333-3

**Published:** 2016-04-05

**Authors:** Joanne M. Heraty, Norman C. Ellstrand

**Affiliations:** Department of Plant Sciences, University of California, Davis, CA USA; Department of Botany and Plant Sciences, University of California, Riverside, CA USA

**Keywords:** *Zea mays*, biodiversity conservation, urban agriculture, germplasm erosion, genetic resources, crop evolution, home gardens, community gardens, immigrant farmers, ethnobotany

## Abstract

Contemporary germplasm conservation studies largely focus on *ex situ* and *in situ* management of diversity within centers of genetic diversity. Transnational migrants who transport and introduce landraces to new locations may catalyze a third type of conservation that combines both approaches. Resulting populations may support reduced diversity as a result of evolutionary forces such as genetic drift, selection, and gene flow, yet they may also be more diverse as a result of multiple introductions, selective breeding and cross pollination among multiple introduced varietals. In this study, we measured the amount and structure of maize molecular genetic diversity in samples collected from home gardens and community gardens maintained by immigrant farmers in Southern California. We used the same markers to measure the genetic diversity and structure of commercially available maize varieties and compared our data to previously reported genetic diversity statistics of Mesoamerican landraces. Our results reveal that transnational dispersal creates an opportunity for the maintenance of maize genetic diversity beyond its recognized centers of diversity.

## Introduction

Conserving landraces and crop wild relatives is crucial for maintaining genetic resources for future crop improvement (e.g., Ford–Lloyd et al. 2011; Maxted et al. 1997, 2012b). Landraces and crop wild relatives provide useful genetic material for breeding modern improved lines, minimizing the vulnerability of inbred crops to pathogens and pests, improving performance, and incorporating unique traits (e.g., Lopes et al. 2015; Mano and Omori 2007). Yet the sustainability of landraces is at risk, in both developing and developed countries. In developing countries, critical threats to genetic agrobiodiversity are policies and programs that encourage replacement of landraces with modern cultivars or encouraged migration to cities, often resulting in the abandonment of farming altogether (Nabhan 1985).

Maize genetic diversity in Mexico is eroding both in terms of the number of extant landraces as well as the amount of allelic diversity within creolized varieties (Dyer et al. 2014; van Heerwaarden et al. 2009a). Despite controversy regarding the speed of the decline (Brush et al. 2015; Dyer et al. 2015), “the question no longer is whether genetic erosion remains a presumption but how to respond to it” (Dyer et al. 2015).

Conservation strategies for minimizing germplasm loss in centers of diversity include both *ex situ* and *in situ* methods (Altieri and Merrick 1987; Brown et al. 1989; Brush 1991, 1995; Nabhan 1985; Oldfield and Alcorn 1987). *Ex situ* conservation in gene banks, botanical gardens, and reserves provides a controlled environment to conserve crop genetic resources. However, landraces and their wild and weedy ancestors are often underrepresented in such collections (Crop Wild Relatives and Climate Change 2013; Hammer 2004). *Ex situ* preservation also prevents evolutionary adaptive responses to abiotic and biotic changes (Maxted et al. 2012a; Simmonds 1962). In contrast, *in situ* or farmer–based conservation incorporates traditional agricultural breeding practices that permit crop evolution and adaptation to changing environments while preserving biological material and the social processes connected to them (Altieri and Merrick 1987; Brush 1995). However, *in situ* conservation is often difficult to sustain in the face of socioeconomic forces promoting the replacement of landraces with alternate varieties or crops, especially in developing countries (Frankel 1974; International Board for Plant Genetic Resources 1985; Maxted et al. 2012b; Oldfield and Alcorn 1987). Despite these difficulties, the consensus is that *ex situ* germplasm conservation should be complemented with an *in situ* farmer–based approach (Brush 1995; Hawkes et al. 2000; Maxted et al. 2002).

What has been largely ignored in previous studies of farmer–based germplasm conservation is whether human migration may provide a third opportunity for crop genetic diversity maintenance. At times, transnational migrants transport crop seeds from their homeland across international borders (e.g., Perales et al. 2003; Soleri et al. 2005). If they plant those seeds and introduce threatened varieties to new locations, they may create conditions for a unique kind of germplasm conservation that combines both *in situ* and *ex situ* approaches. Traditional crops, like maize, managed by migrant farmers in Southern California’s urban gardens mimic *in situ* conservation in that the traditional practices are built on first–hand knowledge of traits, management, and informal breeding techniques. Because those crops are not located in centers of diversity or in centers of crop origin, they are removed from the geographic foci of recognized *in situ* conservation. Maize cultivated by migrant farmers in Southern California’s urban gardens supports a novel environment in that their immediate origin parallels *ex situ* grow–out conditions.

Whether the crops of a diaspora warrant conservation attention depends on whether they harbor relatively high or unique diversity. The amount of genetic diversity in anthropogenically dispersed landraces depends on the combined action of the evolutionary forces of genetic drift, selection, and gene flow (Hancock 2004). Genetic drift via founder effect due to limited initial seed samples, bottlenecks, reduced gene flow from other sources, and/or chronically small crop population size results in depleted diversity. Likewise, strong selection for adaptation to new conditions can reduce genetic diversity. Migrants have made a deliberate effort to collect and transport genetically diverse maize seeds, the diversifying selection of which may lead to urban garden plots that are as much or more genetically diverse than source populations. Furthermore, gene flow in the form of repeated introduction of seeds from the same or different source populations, seed exchange among farmers, and cross–pollination between adjacent plots would be expected to increase diversity (e.g., van Heerwaarden et al. 2009b). Despite these varied expectations, to our knowledge, the relative within–population diversity of anthropogenically dispersed crops has not been studied.

Southern California provides an exemplary case study of joint human and plant migration. California immigrants from Mexico and Mesoamerica frequently maintain plots of crops from their homelands in urban home gardens and community gardens. Home gardens are typically small, multi–species agroecosystems located near their associated household (Galluzzi et al. 2010), whereas community gardens are characteristically larger in size and composed of individual plots with additional common areas where garden meetings and other social events take place. Such gardens have recently been hypothesized to serve as reservoirs of genetic as well as taxonomic diversity (Altieri and Merrick 1987; Corlett et al. 2003; Galluzzi et al. 2010; Hodgkin 2002; Hoogendijk and Williams 2002; Watson and Eyzaguirre 2002; Zaldivar et al. 2004).

In this study, we compared maize genetic diversity (*Zea mays* spp. *mays*) sampled from Southern Californian home gardens and community gardens to local commercial varieties for sale. Likewise, we used genetic diversity data for Mesoamerican maize reported in the literature to determine whether the gardens were more or less diverse relative to those in centers of diversity. We chose maize because the genetic erosion of maize diversity in Mexico, its center of domestication and diversity, has gained increased attention as farmers abandon the cultivation of traditional landrace varieties either in favor of migration to urban centers or in favor of relatively homogenous improved lines. Our results provide an examination of the population genetics of maize in Southern California gardens and reveal that maintaining maize germplasm diversity is not restricted to its traditionally recognized centers of diversity.

## Materials and Methods

### Plant Material

**Fig. 1:**
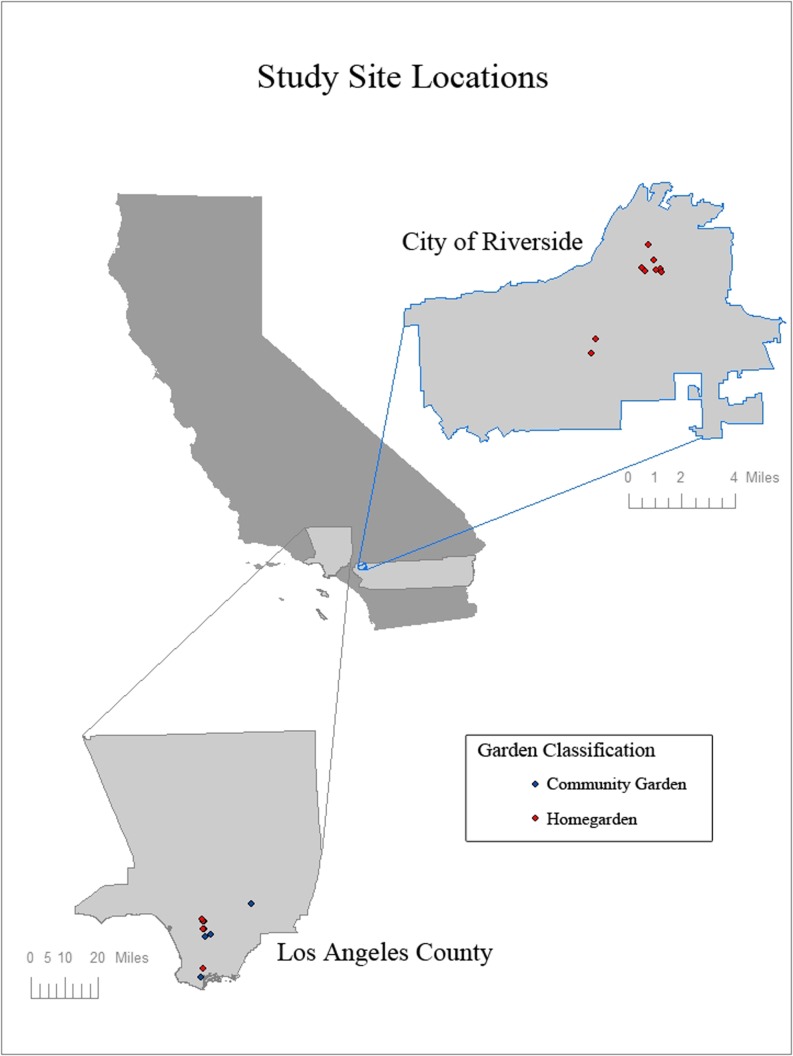
Geographic map of locations of maize samples collected from home garden and community gardens in California. Credit L. M. Hayden.

We collected maize samples between June and September 2008 in Los Angeles and Riverside, California. Populations and participants were selected at random in Mesoamerican enclave communities by visually surveying urban gardens in Los Angeles and Riverside and selecting households and gardens cultivating maize and willing to participate in the experimental study. Twenty total locations were sampled: six home gardens and four community gardens in Los Angeles and ten home gardens in Riverside. Locations of sampled populations are shown in Fig. 1. Table 1 gives detailed collection information for each site.

**Table 1:** Results from PopGene analysis by maize population sampled from home gardens, community gardens, and commercial varieties including collection ID, location, and number of accessions collected.^*^

Population #	Classification ID	Classification	Location	# of accessions	# of polymophic loci	P	N_a_	Mean N_a_	StDev N_a_	H_e_	StDev H_e_	F_is_
1	RIHG1	HG	Riverside, CA	10	5	1.00	16	3.20	1.79	0.53	0.23	0.05
2	RIHG2	HG	Riverside, CA	10	4	0.80	10	2.00	0.71	0.37	0.22	−0.15
3	RIHG3	HG	Riverside, CA	10	5	1.00	13	2.60	0.89	0.45	0.21	0.12
4	RIHG4	HG	Riverside, CA	10	5	1.00	15	3.00	1.22	0.48	0.03	0.00
5	RIHG5	HG	Riverside, CA	10	5	1.00	11	2.20	0.45	0.47	0.04	0.08
6	RIHG6	HG	Riverside, CA	10	4	0.80	13	2.60	1.14	0.35	0.22	−0.21
7	RIHG7	HG	Riverside, CA	10	5	1.00	12	2.40	0.55	0.57	0.95	−0.08
8	RIHG8	HG	Riverside, CA	10	5	1.00	14	2.80	2.15	0.56	0.05	0.06
9	RIHG9	HG	Riverside, CA	10	5	1.00	13	2.60	0.89	0.47	0.26	0.07
10	RIGH10	HG	Riverside, CA	10	5	1.00	12	2.40	0.55	0.32	0.18	−0.16
11	LAHG1	HG	Los Angeles, CA	10	5	1.00	10	2.00	0.00	0.38	0.12	0.23
12	LAHG2	HG	Los Angeles, CA	10	5	1.00	13	2.60	0.55	0.45	0.25	0.10
13	LAHG3	HG	Los Angeles, CA	10	5	1.00	17	3.40	2.32	0.57	0.14	0.22
14	LAHG4	HG	Los Angeles, CA	10	4	0.80	10	2.50	0.58	0.47	0.10	0.00
15	LAHG5	HG	Los Angeles, CA	10	4	0.80	11	2.20	1.10	0.45	0.28	−0.08
16	LAHG6	HG	Los Angeles, CA	10	4	0.80	10	2.00	0.71	0.41	0.24	0.61
17	LAHG7	CG	Los Angeles, CA	10	5	1.00	10	2.00	0.00	0.39	0.18	−0.40
18	LACG1	CG	Los Angeles, CA	10	2	0.40	6	1.50	0.58	0.26	0.30	−0.14
19	LACG2	CG	Los Angeles, CA	10	4	0.80	9	1.80	0.45	0.25	0.19	0.28
20	LACG3	CG	Los Angeles, CA	10	5	1.00	16	3.20	0.84	0.55	0.11	0.21
21	LACG4	CG	Los Angeles, CA	10	4	0.80	9	1.80	0.45	0.24	0.20	0.13
22	LACG5	CG	Los Angeles, CA	10	5	1.00	15	3.00	1.00	0.56	0.15	0.34
23	HV1	HV		10	2	0.40	7	1.75	0.96	0.21	0.30	0.63
24	HV2	HV		10	1	0.20	6	1.20	0.45	0.04	0.08	1.00
25	IV1	IV		10	0	0.00	5	1.00	0.00	0.00	0.00	0.00
26	IV2	IV		10	2	0.40	7	1.40	1.32	0.19	0.27	−0.67
27	SV1	SV		10	4	0.80	11	2.20	0.84	0.40	0.23	−0.32

^*^Key: HG, home garden; CG, community garden; HV, hybrid variety; IV, industrial variety; SV, supermarket variety. Number of polymorphic loci, P, proportion of polymorphic loci; N_a_, observed number of alleles (including mean±SD); H_e_, mean expected heterozygosity ±SD; F_is_, fixation index are reported.

Interviews were conducted with farmers at each site and are part of a larger ethnographic study exploring the origins, seed networks, and traditional agricultural practices of maize cultivation in these gardens. Details regarding those interviews and the associated methodology are available in Heraty (2010).

We sampled fresh leaf tissue from 10 plants per accession. In two cases (LAHG2, LAHG3 and LACG1, LACG2), respondents distinguished two distinct varieties of maize within their garden. In these instances, 10 plants from each of the two varieties were sampled and treated as separate accessions. Thus, we sampled 10 populations and 10 accessions from Riverside and 10 populations and 12 accessions from Los Angeles. Fresh leaf tissue was frozen at −80°C until DNA extraction could be performed.

We included five commercially available cultivars for comparison. Two sweet corn populations (HV1 and HV2) that are representative of locally available horticultural varieties for home gardeners were purchased from a Riverside garden retail store. We also obtained imported corn seed for human consumption from Mexico from a bulk food bin at Big Saver Foods supermarket in Riverside. The latter collection was motivated by interviews with respondents that revealed local ethnic markets supplying bulk seed for consumption were sometimes a source of seeds for their gardens. Finally, we included maize cultivars IV1 and IV2 to represent industrial maize cultivars. We germinated 10 randomly selected seeds from each cultivar in a temperature–controlled greenhouse. Fresh leaf tissue was harvested from germinated plants and stored at −80°C prior to extraction.

DNA was isolated from finely ground tissue by use of the DNeasy plant Mini Kit (Qiagen, Valencia, CA) with the following modifications: 3 μl Rnase A stock solution was added to the ground tissue; at the lysation phase, cells were incubated for 15 min; and at the final elution step, 50 μl Buffer AE was added to the DNeasy membrane. Extracted DNA was quantified with use of NanoDrop spectrophotometer (Thermo Fisher Scientific, Ealtham, MA) and stored at −20°C.

### PCR (Polymerase Chain Reaction) Amplification

Five microsatellite markers previously described by Senior et al. (1998) were selected for analysis. The selected primer sequences are listed and detailed in Table 2. More information for each primer set can be found at http://www.maizegdb.org.

**Table 2:** Primer information.

MARKER	CHROMOSOME	REPEAT	PRIMER SEQUENCE	Min/MAX Allele
Phi079	4	AGATG	TGGTGCTCGTTGCCAAATCTACGA	179/196
			GCAGTGGTGGTTTCGAACAGACAA	
Phi115	8	AT/ATAC	GCTCCGTTTTCGCCTGAA	291/312
			ACCATCACCTGAATCCATCACA	
Phi102228	3	AAGC	ATTCCGACGCAATCAACA	122/131
			TTCATCTCCTCCAGGAGCCTT	
Phi427424	2	ACC	CAACTGACGCTGATGGATG	123/139
			TTGCGGTGTTAAGCAATTCTCC	
Phi093	4	AGCT	AGTGCGTCAGCTTCATCGCCTACAAG	283/295
			AGGCCATGCATGCTTGCAACAATGGATACA	

DNA amplification involved the use of a PTC–100 Thermo Cycler (MJ Research Inc., Watertown, MA) in a 10 μl volume containing approximately 5.0 to 10.0 ng/μl template DNA, 10 μm each forward and reverse primer, 1 mM dNTPs, 10x Promega PCR Buffer, 25 μm magnesium chloride, and 5 units/μl Taq DNA polymerase. PCR conditions were 94°C for 5 min, 34 cycles of 94°C for 1 min, 55°C for 4 min, 72°C for 2.5 min, and a final cycle of 72°C for 15 min.

### Polymorphism Detection

Polymorphisms in the amplified PCR product were detected by electrophoresis in a 2% Agarose Super Fine Resolution (SFR) gel (Amresco, Solon, OH). Gels were prepared with 1X TBE Buffer and were stained with ethidium bromide before electrophoresis in a submarine gel system (Wide Mini–sub Cell GT, Bio–Rad, Hercules, CA). Each well contained 4 μl PCR product and 1 μl 6X blue loading dye. Bands were visualized by use of GelDOC (Bio–Rad, Hercules, CA). Fragments were estimated by comparison with a 100–bp DNA ladder (New England Biolabs, Ipswich, MA). Each gel was run with a control sample of a known fragment size.

DNA for several individuals failed to amplify at certain loci, despite repeated amplifications, particularly B73. These data are treated as missing in our analyses.

### Data Analysis

For each locus, bands were scored as heterozygous or homozygous, depending on the presence of a single or double band. Allele frequencies were calculated for each accession. Observed number of alleles (N_a_), proportion of polymorphic alleles, percentage of polymorphic loci, expected heterozygosity (H_e_), and fixation index (F_is_) were calculated for each population and population group by use of the computer program PopGene v1.32 (Yeh et al. 1997).

**Fig. 2:**
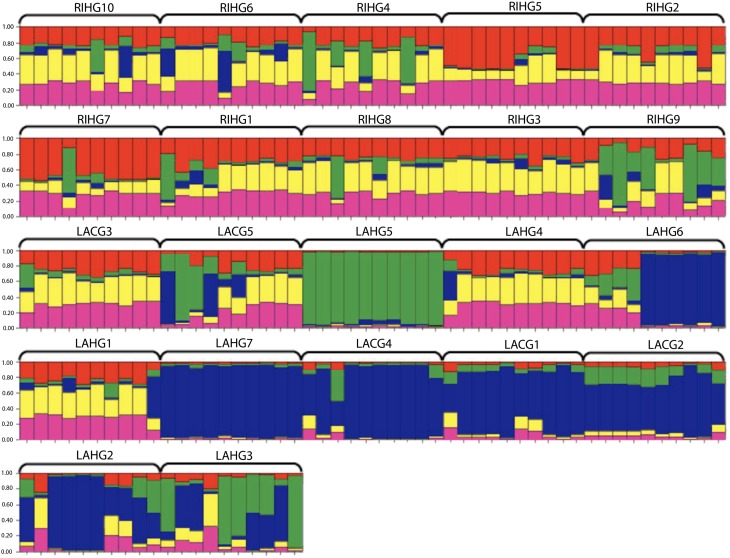
STRUCTURE analysis of garden populations of maize. Most likely grouping K=5.

**Fig. 3:**
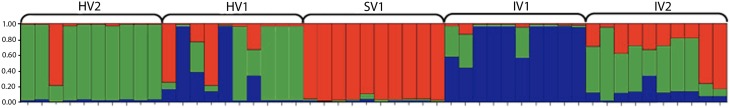
STRUCTURE analysis of commercial populations of maize. Most likely grouping K=3.

**Fig. 4:**
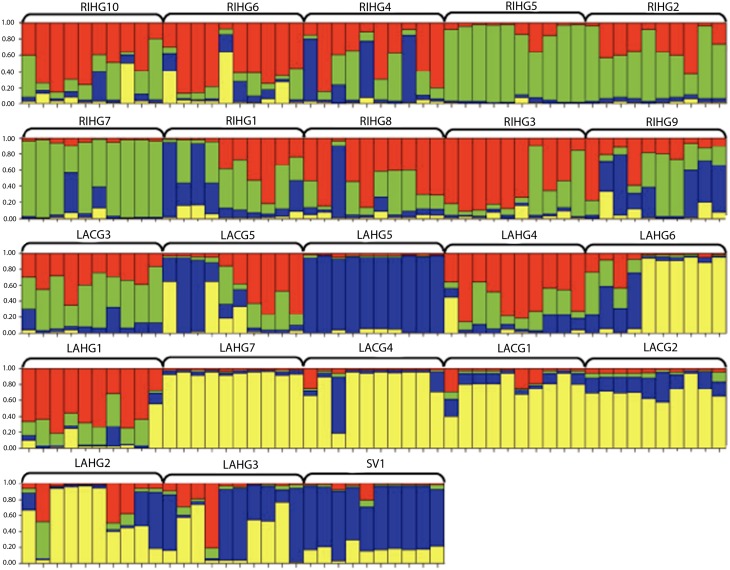
STRUCTURE analysis of garden populations of maize including imported supermarket variety SV1. Most likely grouping K=4.

Microsatellite data were also analyzed for population genetic structure by use of STRUCTURE v2.2 (Pritchard et al. 2000) with a burn–in of 100,000 iterations run against a simulation of 250,000 iterations to estimate the genetic cluster (K) parameters. The optimal K–value can be estimated by graphing the output information for the log–transformed probability of K clusters [lnPr(X∣K)] from each run to display the greatest decrease in slope. Multiple runs at different K–values were conducted for accurate results. We conducted three separate analyses using STRUCTURE: one with all garden populations (Fig. 2), a second with all commercial populations (Fig. 3), and a third that assigned the imported bulk bin seed from Mexico to the garden populations group (Fig. 4).

## Results

Microsatellite polymorphisms were detected for every locus. Population genetic properties for each individual population are in Table 1. Table 3 reports the distribution of genetic variation according to source: garden populations versus commercial populations, and commercial populations without the imported supermarket variety SV1. Table 3 also reports the distribution of genetic variation in garden populations by home gardens versus community gardens.

**Table 3:** Results from PopGene analysis separated by garden populations (including a subanalysis between home gardens and community gardens) and commercial populations (including a separate analysis removing the imported supermarket variety SV1).

	N_a_	Mean N_a_	StDev N_a_	# of Polymorphic Loci	P	H_e_	StDev H_e_	F_is_
Garden Populations	25	5.00	1.87	5	1.00	0.55	0.11	0.05
**Homegardens	25	5.00	1.87	5	1.00	0.55	0.10	0.05
**Community Gardens	19	3.80	1.30	5	1.00	0.51	0.16	0.09
Commercial Populations	14	2.80	1.10	4	0.80	0.31	0.29	−0.23
Commercial Populations without SV1	9	1.80	0.84	3	0.60	0.19	0.22	0.06

^*^Key: SV, supermarket variety. Number of polymorphic loci, P, proportion of polymorphic loci; N_a_, observed number of alleles (including mean±SD); H_e_, mean expected heterozygosity ±SD; F_is_, fixation index are reported.

All garden populations were polymorphic (100%). The total number of alleles observed was 25 (mean 5.00±1.87). The mean expected heterozygosity (H_e_) for the group was 0.55±0.11, with mean F_is_ for the group of 0.05. A single population, LACG1 (one accession of two sampled from a Los Angeles community garden), had the lowest number of observed alleles, 6, and LAHG3 (one accession of two sampled from a Los Angeles home garden) had the highest, 17.

In contrast, commercial populations showed less genetic diversity than garden populations, regardless of population genetic measurement. Only four loci were polymorphic (80%). The total number of observed alleles was 14 (mean 2.80±1.10). The mean H_e_ was 0.31±0.29 with mean F_is_ for the group of −0.23. The population B73 had the lowest number of observed alleles, 5. Interestingly, the population SV1 (supermarket variety of imported Mexican seed) had the highest number of total alleles, 11. Two–tailed *t*–test revealed significant difference in diversity (H_e_) of individual garden populations and individual commercial populations (including SV1) (p≤0.016). When we removed the imported SV1 variety from the commercial group statistics, the total number of alleles for the commercial populations decreased to 9 (mean 1.80±0.84). The mean H_e_ was 0.19±0.22, with the mean F_is_ for the group of 0.06.

The contrast between home and community gardens was not nearly as strong. Both garden types were polymorphic at all five loci (100%). The total number of alleles in home gardens was 25 (mean 5.00±1.87), with 19 (mean 3.80±1.80) in community gardens. The mean H_e_ for home gardens was 0.55±0.10; for community gardens and 0.51±0.10 for community gardens. The mean F_is_ for home gardens was 0.05 and 0.09 for community gardens.

The program STRUCTURE assigned genetic affiliations to the sampled individuals within preselected populations. The most likely number of groups for all garden populations (unresolved amplifications treated as missing) was K = 5 (Fig. 2). Generally, most populations showed a moderate to high degree of admixture, except for population LAHG5, which grouped independently and uniformly from other garden populations. Also, populations LAHG6, LAHG7, LACG4, LACG1, LACG2, and LAHG2 showed some affiliation with each other.

Separate analysis of the five commercial varieties gave a group assignment of K = 3 (Fig. 3). The population SV1 (supermarket variety of imported Mexican seed) showed little genetic affiliation to other commercial populations. We reassigned SV1 to the garden populations to explore whether it had any affinity with them. Interestingly, adding SV1 to the garden populations gave a group assignment of K = 4 (Fig. 4). Furthermore, SV1 showed some genetic affiliation to some garden populations, especially LAHG5.

## Discussion

As compared with commercially available maize from Southern California, maize collected from garden populations in Southern California showed a considerable amount of genetic variation in all five microsatellite loci investigated (Table 3). The results of this analysis assume that the samples of both the gardens and commercial populations are representative of the range of diversity of maize in Southern California. Several varieties of both cultivated and commercial maize could have served as comparative populations and varieties used in this study are assumed to be representative of overall varieties.

This analysis of the structure of diversity in the gardens gave several interesting results. First, PopGene revealed greater relative diversity in gardens than in commercial populations. In the STRUCTURE analysis, the imported SV1 corn seed showed greater allelic similarity to maize sampled from gardens than any of the commercial varieties, so the garden populations may have a closer genetic affiliation to this imported seed from Mexico. PopGene analysis showed less overall diversity in the commercial populations sampled.

Results of STRUCTURE analysis showed that the gardens exhibited greater admixture within and between populations than the commercial populations. While these results may indicate potential gene flow, it may also indicate a high degree of relatedness due to similar ancestry. When analyzing the commercial populations alone, SV1, the imported seed from Mexico, did not pair with the rest of the commercial varieties. The store labeling of this seed as an imported corn seed from Mexico suggests a potential landrace origin; if so, our finding of little genetic affiliation with other locally available commercial varieties is not surprising. On analyzing SV1 with the garden populations, SV1 showed a common affiliation with the populations LAHG5, LAHG7, LACG4, LACG1, LACG2, LAHG2, and LAHG3. The resulting affiliation suggests that the farmers who managed the populations might have used the supermarket variety in the past as a seed source or that some of the garden accessions share ancestry with the imported seed.

We considered that a community garden with multiple plots and accessions might have increased potential for gene flow and admixture, thus showing a more diverse germplasm profile. Results from grouped PopGene analysis of home garden and community garden populations do not support our original assumption of greater variation. These results could be attributed to the small number of community gardens we sampled as compared with the large number of home garden populations. Sampling from a larger set of community gardens in future studies would shed light on whether a small sample size affects the comparative outcome or whether other factors are involved.

Several existing studies have reported on the genetic diversity of landraces of maize in Mexico. Their results (Table 4) on fixation index, allele number, and expected heterozygosity serve as comparison studies to our data if samples from the home gardens and community gardens in Southern California have purported landrace ancestry. Overall, the levels of garden maize genetic diversity and their organization are approximately similar to what has been found in population genetic analyses of landraces in Mesoamerica.

**Table 4:** Published Mesoamerican maize diversity statistics used for comparative analysis.

Authors	Type of Marker	No. SSRs	No. Zea mays L. ssp. mays Accessions	No. of Plants Genotyped	Source	Total No. Alleles	Mean Gene Diversity	Mean Fixation Index
Doebley et al. (1985)	Isozyme	23 loci	94	12 per accession	Mexico/Guatemala	163 (range 3-18)	0.182 (range 0.01-0.60)	N/A
Heraty and Ellstrand, unpubl. Data	SSR	5.00	22	10 per accession	Home Gardens and Community Gardens in Southern California	Home Gardens (HG: 25 total) Community Gardens (CG: 19 total)	HG: 0.55±0.10 CG: 0.51±0.16	HG: 0.05 CG: 0.09
Pressoir and Berthaud (2004)	SSR	11.00	20	1	Mexico	N/A	0.71 (range 0.65-0.72)	0.138±0.031
Reif et al. (2006)	SSR	25.00	25	497 (total)	Mexico/Guatemala	196 (range 4-14)	0.61	0.24 (range 0.09-0.47)
Sanchez et al. (2000)	Isozyme	37 loci	209	12 per accession	Mexico	303 (range 59-120)		N/A
Vigouroux et al. (2008)	SSR	96	945	964	North America, Central America, South America	3752 Averages within 4 Clusters: Highland Maize (HM: 14.9) Tropical Lowland (TL:14.4) Andean (A: 12.4) Northern US (NUS: 10.6)	HM: 0.81±0.01 TL: 0.80±0.01 A: 0.71±0.02 NUS: 0.72±0.02	HM: 0.33 TL: 0.331 A: 0.29 NUS: 0.44
Warburton et al. (2011)	SSR	17	59	15 per accession	Mexico/Guatemala	N/A	0.481±0.03 (within landraces from Mexico/Guatemala)	N/A

Our findings are unique for this area of study. Existing research into maize biodiversity conservation has focused on farmer–based methods of biodiversity conservation of maize in centers of crop diversity and *ex situ* methods to preserve diversity in controlled environments. It has ignored the importance of human migration and the movement of important cultural food species in establishing new regions of germplasm diversity. Biodiversity research of urban gardens worldwide has been limited in two ways. Geographically, studies have mostly concentrated on tropical home gardens in Asia and South America. Research has also been limited by its focus on species richness and evenness within gardens without addressing the level of genetic diversity within or between species (see Aguilar–Støen et al. 2009; Coomes and Ban 2004; Lamont et al. 1999; Padoch and De Jong 1991; Soemarwoto and Conway 1992; Thompson et al. 2003).

Our study provides compelling evidence that, as compared with available commercial varieties of maize, populations of maize from home gardens and community gardens in Southern California show large variations in genetic diversity both within and between populations. Our comparison with literature–sourced landrace studies provided in Table 4 reveals that urban gardens may serve as underappreciated locations of *ex situ* diversity managed *in situ*.

Our study encourages an expansion of research investigating the biogeography of diversity in urban gardens throughout North America and the mechanisms by which migrating farmers select and maintain landraces and crop wild relatives. With the disruption of farming communities and maize diversity in Mexico, immigrants who relocate transnationally have become important stewards in introducing, conserving, and actively managing diverse maize germplasm, ensuring that it finds refuge in new environments where it can continue to evolve and adapt.

## Acknowledgements

This research was supported by a National Science Foundation Biocomplexity Grant (DEB–0409984) and a UCMEXUS–CONACYT Collaborative Research Grant awarded to NCE. Cristina Martinez–Canton provided invaluable field support. Laura Smales, Jeffrey Ross–Ibarra, Daniela Soleri, and two anonymous reviewers provided helpful comments to improve earlier drafts of this manuscript.
